# Effect of SARS-CoV-2 mRNA-Vaccine on the Induction of Myocarditis in Different Murine Animal Models

**DOI:** 10.3390/ijms24055011

**Published:** 2023-03-06

**Authors:** Vanessa A. Zirkenbach, Rebecca M. Ignatz, Renate Öttl, Zeynep Cehreli, Vera Stroikova, Mansur Kaya, Lorenz H. Lehmann, Michael R. Preusch, Norbert Frey, Ziya Kaya

**Affiliations:** 1 Department of Cardiology, University of Heidelberg, 69120 Heidelberg, Germany; 2 DZHK (German Centre for Cardiovascular Research), Partner Site Heidelberg/Mannheim, University of Heidelberg, 69120 Heidelberg, Germany

**Keywords:** SARS-CoV-2, mRNA vaccine, side effects, myocarditis, myocardial function

## Abstract

In the course of the SARS-CoV-2 pandemic, vaccination safety and risk factors of SARS-CoV-2 mRNA-vaccines were under consideration after case reports of vaccine-related side effects, such as myocarditis, which were mostly described in young men. However, there is almost no data on the risk and safety of vaccination, especially in patients who are already diagnosed with acute/chronic (autoimmune) myocarditis from other causes, such as viral infections, or as a side effect of medication and treatment. Thus, the risk and safety of these vaccines, in combination with other therapies that could induce myocarditis (e.g., immune checkpoint inhibitor (ICI) therapy), are still poorly assessable. Therefore, vaccine safety, with respect to worsening myocardial inflammation and myocardial function, was studied in an animal model of experimentally induced autoimmune myocarditis. Furthermore, it is known that ICI treatment (e.g., antibodies (abs) against PD-1, PD-L1, and CTLA-4, or a combination of those) plays an important role in the treatment of oncological patients. However, it is also known that treatment with ICIs can induce severe, life-threatening myocarditis in some patients. Genetically different A/J (most susceptible strain) and C57BL/6 (resistant strain) mice, with diverse susceptibilities for induction of experimental autoimmune myocarditis (EAM) at various age and gender, were vaccinated twice with SARS-CoV-2 mRNA-vaccine. In an additional A/J group, an autoimmune myocarditis was induced. In regard to ICIs, we tested the safety of SARS-CoV-2 vaccination in PD-1^−/−^ mice alone, and in combination with CTLA-4 abs. Our results showed no adverse effects related to inflammation and heart function after mRNA-vaccination, independent of age, gender, and in different mouse strains susceptible for induction of experimental myocarditis. Moreover, there was no worsening effect on inflammation and cardiac function when EAM in susceptible mice was induced. However, in the experiments with vaccination and ICI treatment, we observed, in some mice, low elevation of cardiac troponins in sera, and low scores of myocardial inflammation. In sum, mRNA-vaccines are safe in a model of experimentally induced autoimmune myocarditis, but patients undergoing ICI therapy should be closely monitored when vaccinated.

## 1. Introduction

Since the novel coronavirus started spreading around the globe in 2019, many millions of people have become infected, and the virus has developed into a global pandemic, causing enormous health and socioeconomic damage [1,2]. SARS-CoV-2 is transmitted via respiratory particles, and shows a wide range of symptoms, including fever, dry cough, shortness of breath, anorexia, and fatigue. In serious cases, respiratory support is needed [3,4]. The rapid increase of infected people requiring hospitalization led to an overload of hospitals in many countries, and particularly, elderly people with low immunity were at high risk [5]. In order to fight back against SARS-CoV-2, research into potential vaccines has been conducted worldwide. One novel class of vaccines, which was developed by the pharmaceutical companies BioNTech/Pfizer and Moderna, is based on immunization with a modified mRNA that encodes for the SARS-CoV-2 spike protein [6,7,8,9]. After entering the human body, endogenous cells produce the viral spike protein and present it to immune cells. These cells pass on an immune reaction with specific antibody production. To transport the vaccine into the cell, lipid nanoparticle (LNP) structures are used. These spherical vesicles consist of ionized lipids that can bind cells by their charge, and thus introduce their contents into the cells by endocytosis [6,8,9,10,11]. As one of the post-vaccination side effects, myocarditis has been documented more frequently [12,13,14,15]. However, recently published clinical studies showed a low incidence for myocarditis, but this side effect mainly cumulates in young men after the second vaccination [16,17,18]. In humans, both age and gender seem to play a role in the development of an autoimmune reaction after a SARS-CoV-2 vaccination [19,20]. So far, the exact pathomechanism is not understood. Potential processes might be epitope spreading, or cross-reactivity between SARS-CoV-2 antigens and a variety of tissue antigens, some of which are located in the cardiovascular system [21,22]. Accordingly, Mouch et al. reported symptoms of chest pain/discomfort, mainly after the second vaccination with the BioNTech vaccine, in six male patients in an age range between 16 and 45 years (median 22 years). All six patients showed abnormal electrocardiograms and elevated serum C-reactive protein levels, as well as high-sensitivity cardiac troponin T or cardiac troponin I levels [23]. Some retrospective studies, mostly using register data, describe an association between pre-existing autoimmune conditions and an increased risk of myocarditis after SARS-CoV-2 vaccination [21,24,25,26]. Myocarditis is an inflammatory disease of the myocardium, and is characterized by the infiltration of mono-nucleated cells [27]. In most cases, myocarditis is self-limiting and easily treatable with symptomatic medication [28,29]. Nevertheless, in worst cases, it can lead to a permanent limitation of cardiac function, and to dilatative cardiomyopathy, which can result in sudden cardiac death [30,31].

Little is known about the direct effect of the genetic background of a species on developing myocarditis after vaccination. Mouse models for examining myocarditis already exist, showing different susceptibilities in developing myocarditis [31,32,33,34,35,36,37,38,39,40]. Furthermore, there is almost no data on the risk and safety of vaccination in patients who are already diagnosed with an ongoing myocarditis from other causes, such as viral myocarditis or autoimmune myocarditis. Currently, the risks of vaccination in combination with other therapies (e.g., ICI therapy), and/or ongoing diseases, are poorly understood. Immunomodulation in cancer therapy is intended to activate the immune system in order to fight the tumor. Therefore, antibodies against programmed cell death protein 1 (PD-1), or cytotoxic T-lymphocyte-associated protein-4 (CTLA-4), are applied, since their activation via binding their respective ligands would otherwise lead to an inhibition of T-cell activity, and down-regulation of the immune response [41]. While this treatment can lead to success in tumor therapy, it can also cause various autoimmune side effects (e.g., myocarditis) by affecting self-tolerance [42]. Since this might also relate to the development of myocarditis, it represents an additional risk factor for vaccination against SARS-CoV-2. Additionally, these groups of patients are usually at higher risk for severe clinical outcome and death when infected with SARS-CoV-2, because of their underlying primary disease, mostly in advanced cancer stages. Therefore, we investigated the potential safety and potential side effects of vaccination in different mouse strains. We vaccinated a mouse strain (A/JOlaHsd) known to be very susceptible for induction of autoimmune diseases, and a mouse strain known to be more resistant, with mRNA-vaccine (BioNTech) [31]. We used mice at different ages and gender to test the age and gender-related effects.

Furthermore, we studied the safety and side effects of vaccination during an ongoing myocarditis in an EAM mouse model. Finally, we studied the vaccination by itself, and in combination with other immune-based therapies that have myocarditis as a side effect, e.g., immune checkpoint inhibition in cancer therapy, in PD-1^+/+^ control mice and PD-1 knockout (PD-1^−/−^) mice [43,44,45,46]. The results might provide a basis for competent risk assessment relating to the side effects and cross-reactions of SARS-CoV-2 vaccination, and the development of future mRNA vaccines for these groups of patients.

## 2. Results

### 2.1. Influence of mRNA-Vaccine Depending on Age and Gender

To investigate the safety and risks of vaccination with mRNA-vaccine against SARS-CoV-2 on the development of myocarditis, A/J mice were first vaccinated with mRNA-vaccine at day 0 and day 21. Since A/J mice are more susceptible for the development of an autoimmune myocarditis, a lower dose of 0.2 µg per injection was initially chosen [31,47]. To examine the potential gender and age specific effect, A/J mice at different ages (5–7 and 23-weeks) and gender were used.

**Figure 1:**
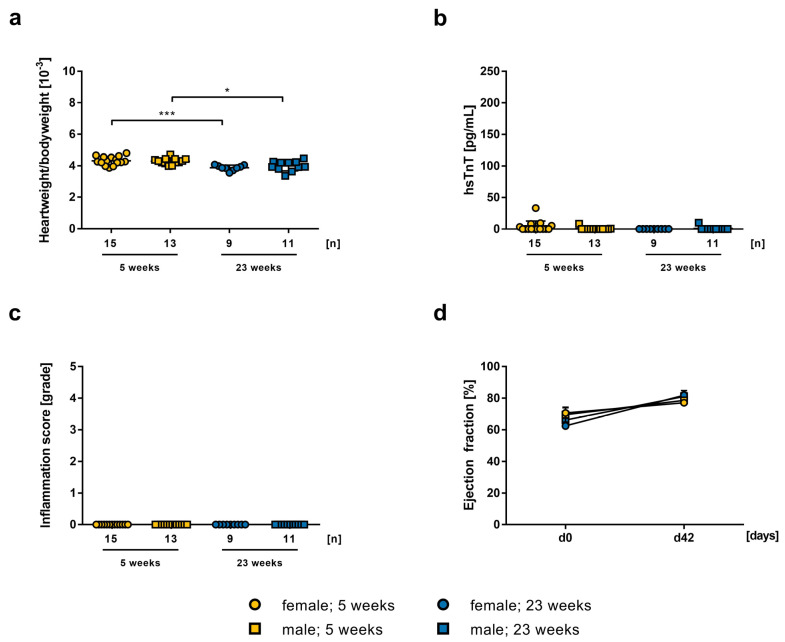
Effect of 0.2 µg mRNA-vaccine on 5–7 weeks old female (n = 15) and male (n = 13), and on 23-weeks old female (n = 9) and male (n = 11) A/J wt mice. Data is presented as a mean ± SEM. (**a**) Calculated heart weight to body weight ratio on day 42. Statistical significance was determined by one-way ANOVA: * *p* = 0.0358, *** *p* = 0.0007. (**b**) Evaluation of hsTnT-levels in mouse serum collected on day 42. (**c**) Histopathological examination of inflammation score in grades in HE stained heart sections on day 42, evaluated by two independent investigators. (**d**) Echocardiographic examination of ejection fraction using M-mode on day 0 and day 42.

The heart weight to body weight ratio in younger females compared to older females (4.36 ± 0.0624, 3.88 ± 0.0558, *p* = 0.0007, respectively) and in younger males compared to older males (4.25 ± 0.0616, 3.996 ± 0.0963, *p* = 0.0358, respectively) was increased (Figure 1a). Determination of hsTnT-levels in serum of 5-weeks old female and male mice (1.80 ± 3.34, 3.31 ± 9.26, n.s., respectively) and of 23-weeks old mice (0.91 ± 3.02, 0 ± 0, n.s., respectively) showed no signs of heart damage (Figure 1b). In histopathological evaluation, we could not find any inflammation in heart tissue sections (Figure 1c). The echocardiographic examination on day 42 indicated no deterioration of cardiac pump function in LVEF of 5-weeks old males and females (78.6 ± 1.405, 77.1 ± 1.535, n.s., respectively) and of 23-weeks old mice (81.13 ± 2.225, 81.87 ± 2.834, n.s., respectively), without significant differences between age and gender (Figure 1d). To sum up these results, we detected no difference between male and female animals at different ages, after vaccination with 0.2 µg mRNA-vaccine.

**Figure 2:**
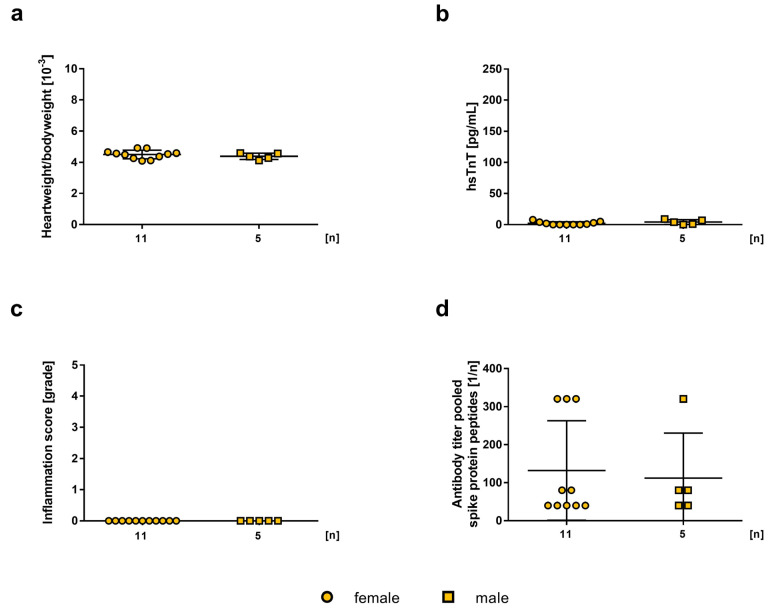
Effect of 2.5 µg mRNA-vaccine on 5-weeks old female (n = 11) and male (n = 5) A/J wt mice. Data is presented as a mean ± SEM. (**a**) Calculated heart weight to body weight ratio on day 42. (**b**) Determination of hsTnT-values in mouse serum collected on day 42. (**c**) Histopathological examination of inflammation score in grades in HE stained heart sections on day 42, evaluated by two independent investigators. (**d**) Determination of produced antibodies against pooled spike protein peptide sequences in mouse serum on day 42 by ELISA.

Next, we tested vaccination with a higher vaccine dosage of 2.5 µg. Neither in male (4.38 ± 0.0878) nor in female (4.50 ± 0.0821) animals treated with mRNA-vaccine did increased heart weight to body weight ratio occur due to connective tissue formation, compared to untreated animals (4.22 ± 0.0872, 4.64 ± 0.198, respectively; Figure 2a). Additionally, there was no significant elevation in hsTnT-levels in male or female mice (4.22 ± 3.823, 1.982 ± 2.533, n.s., respectively; Figure 2b). As observed in 0.2 µg vaccinated mice, no inflammation on a histopathological level could be found after vaccination with 2.5 µg mRNA-vaccine either (Figure 2c). Antibody titers against the spike protein showed no significant differences between females and males (260.0 ± 211.0 vs. 73.33 ± 49.64, n.s., respectively; Figure 2d).

**Figure 3:**
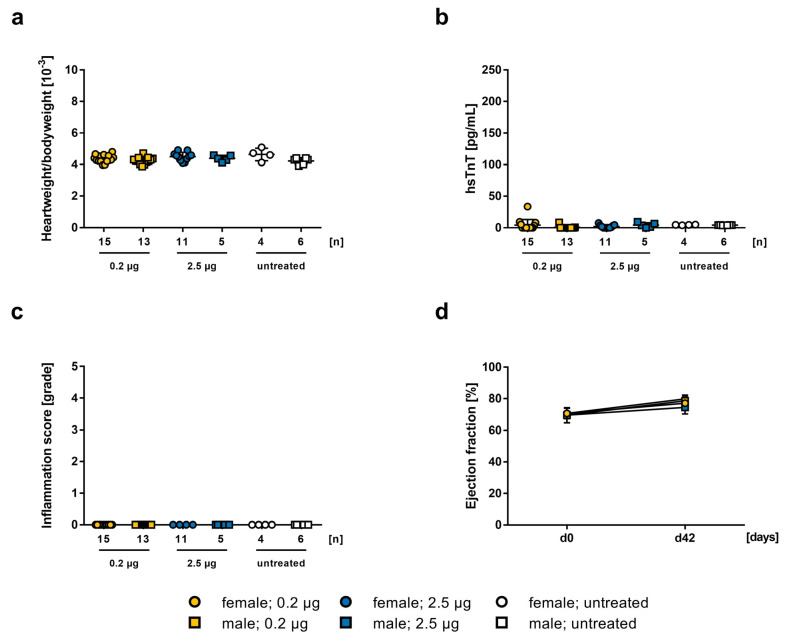
Comparison of 0.2 µg and 2.5 µg mRNA-vaccine on 5-weeks old A/J wt mice with untreated females (n = 4) and males (n = 6). Data is presented as a mean ± SEM. (**a**) Evaluation of heart weight to body weight ratio on day 42. (**b**) Comparison of hsTnT-values in mouse serum collected on day 42. (**c**) Histopathological examination of inflammation score in grades in HE stained heart sections on day 42, evaluated by two independent investigators. (**d**) Echocardiographic examination of ejection fraction using M-mode on day 0 and day 42.

To compare the results of 0.2 µg and 2.5 µg mRNA-vaccine in 5-weeks old A/J mice, both approaches were plotted together, showing no significant dose-dependent effect. Heart weight to body weight ratio of the animals indicated no significant difference between both groups, and compared with untreated control animals (m_0.2_ = 4.248 ± 0.06158, m_2.5_ = 4.384 ± 0.08778, m_untreated_ = 4.217 ± 0.08724, n.s.; f_0.2_ = 4.36 ± 0.06241, f_2.5_ = 4.498 ± 0.08214, f_untreated_ = 4.635 ± 0.198, n.s.; Figure 3a). Serum hsTnT-levels were not pathologically elevated, neither in lower nor in higher dosage of the BioNTech vaccine, in both males (_0.2_ = 39 ± 4.047, _2.5_ = 53 ± 2.227) and females (_0.2_ = 48.69 ± 3.013, _2.5_ = 50.64 ± 1.208; Figure 3b). Histopathological evaluation showed no inflammation in any experimental groups (Figure 3c), and there was no myocardial inflammation or alteration in cardiac function (Figure 3d).

### 2.2. Influence of the mRNA-Vaccine Depending on the Mouse Strain

**Figure 4:**
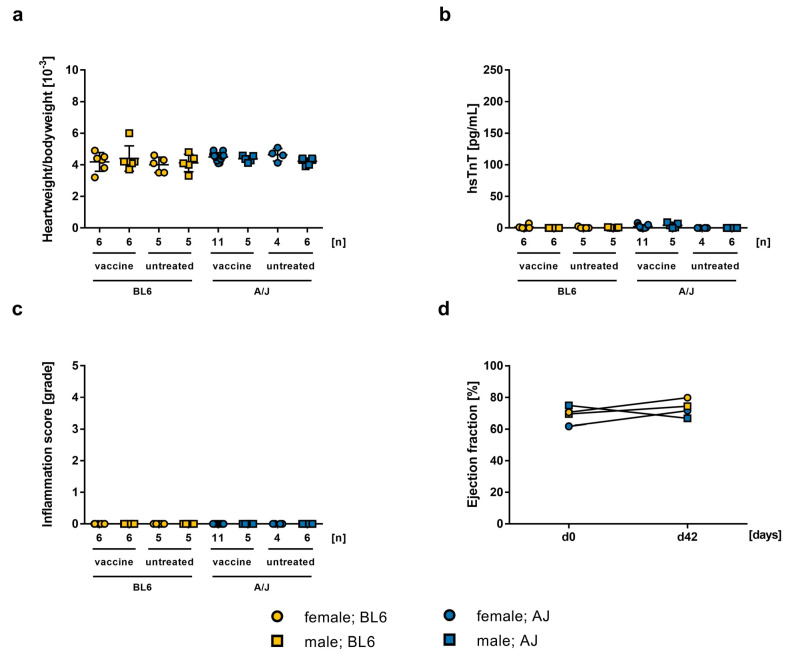
Strain specific differences (BL/6 vs. A/J) of 2.5 µg mRNA-vaccination. Data is presented as a mean ± SEM (vaccine n = 6, untreated n = 5). (**a**) Comparison of heart weight to body weight ratio on day 42. (**b**) Measured hsTnT-levels in mouse sera collected on day 42. (**c**) Histopathological examination of inflammation score in grades in HE stained heart sections on day 42, evaluated by two independent investigators. (**d**) Echocardiographic assessment of ejection fraction using M-mode on day 0 and day 42.

It is well known that mouse strains show different susceptibility in certain disease models [31]. Therefore, we studied two mouse strains that differ in their susceptibility for an induction of EAM. Whereas BL/6 mice are resistant to an induction of experimental myocarditis, A/J mice are very susceptible. Thus, we studied the effect of mRNA-vaccination on BL/6 compared to A/J mice. In both BL/6 female (f_vaccine_ = 4.183 ± 0.2442, f_untreated_ = 4 ± 0.2191, n.s.) and male mice (m_vaccine_ = 45.4 ± 0.3296, m_untreated_ = 4.12 ± 0.2478, n.s.), heart weight to body weight ratio was normal compared to untreated control mice (Figure 4a). Additionally, serum hsTnT-levels did not show signs of cardiac damage (f_vaccine_ = 48.45 ± 2.283, f_untreated_ = 47.12 ± 2.239, n.s.; m_vaccine_ = 43.95 ± 1.041, m_untreated_ = 49.66 ± 1, n.s.; Figure 4b). Histopathological analysis confirmed no signs for inflammation in hearts of BL/6 mice (Figure 4c), and heart function of vaccinated mice was not affected (Figure 4d). Comparing the results of A/J mice treated with 2.5 µg mRNA-vaccine with treated BL/6 mice, no difference was observed. Heart weight to body weight ratio, histology, and hsTnT serum levels were in both cases equally normal, corresponding to the values of untreated animals (Figure 4). Our results suggest that vaccination of both mouse strains has no alteration effect on myocardial function. Even A/J mice, a common model for EAM, showed no susceptibility for the development of myocarditis after SARS-CoV-2 vaccination with the BioNTech mRNA-vaccine.

### 2.3. Influence of mRNA-Vaccination in Combination with Experimental Autoimmune Myocarditis

**Figure 5:**
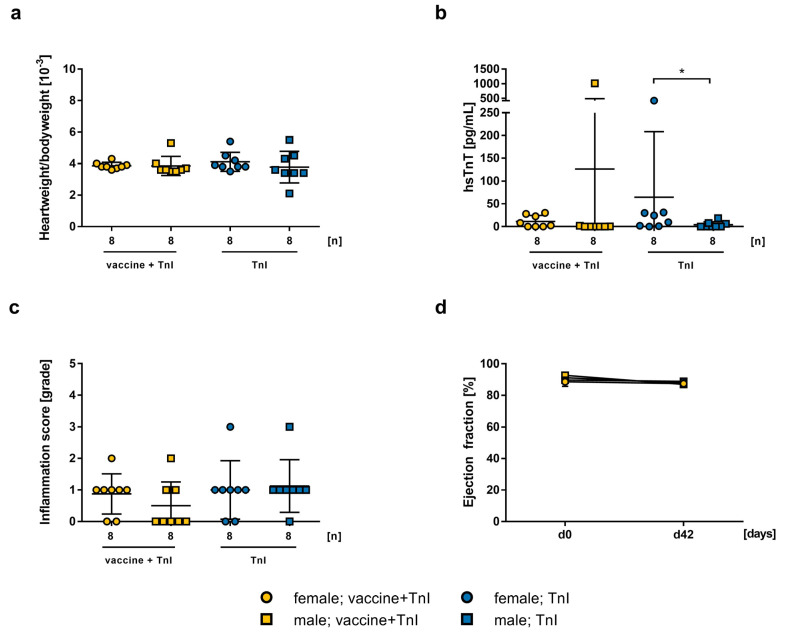
Effect of 2.5 µg mRNA-vaccine on the induction of an experimental autoimmune myocarditis with 150 µg TnI peptide in A/J mice (n = 8). Data is presented as a mean ± SEM. (**a**) Calculated heart weight to body weight ratio on day 42. (**b**) Determination of hsTnT-levels in mouse serum collected on day 42. Statistical significance was determined by Mann–Whitney: * p = 0.0373. (**c**) Histopathological examination of inflammation score in grades in HE stained heart sections on day 42, evaluated by two independent investigators. (**d**) Echocardiographic assessment of ejection fraction using M-mode on day 0 and day 42 after TnI immunization.

To evaluate the effect of additional immunization in a model of autoimmune myocarditis, we vaccinated female and male A/J mice, at the age of 5 weeks, with 2.5 µg mRNA-vaccine twice (on days 0, 21), and simultaneously induced autoimmune myocarditis through immunization with murine cardiac TnI peptide (on days 0, 7, 14). A control group was immunized with TnI peptide without vaccination. To further analyze the effect of gender, we treated and compared both female and male groups. We found no differences in the heart weight to body weight ratio between vaccinated mice and control mice after immunization, neither in female groups (3.863 ± 0.2134, 4.113 ± 0.601, n.s., respectively) nor in male groups (3.85 ± 0.6071, 3.775 ± 1.002, n.s., respectively; Figure 5a). Furthermore, there was no significant difference when comparing hsTnT-levels between females (11.34 ± 13.19, 64.54 ± 143.8, n.s., respectively) and males (126.5 ± 357, 4.088 ± 6.6662, n.s., respectively), but within the group of TnI immunization, the difference between female and male mice was significant (*p* = 0.0373; Figure 5b). To determine cardiac inflammation, analysis of HE stained heart sections showed no significant increase in inflammation in vaccinated females (0.875 ± 0.227, 1 ± 0.328, n.s., respectively) and males (0.5 ± 0.267, 1.125 ± 0.295, n.s., respectively) compared to their respective controls (Figure 5c). There were no significant differences in cardiac function between all groups at any time of measurement (Figure 5d).

### 2.4. Safety of mRNA-Vaccination in PD-1^−/−^ Mice and co-Treatment with CTLA-4 abs

**Figure 6:**
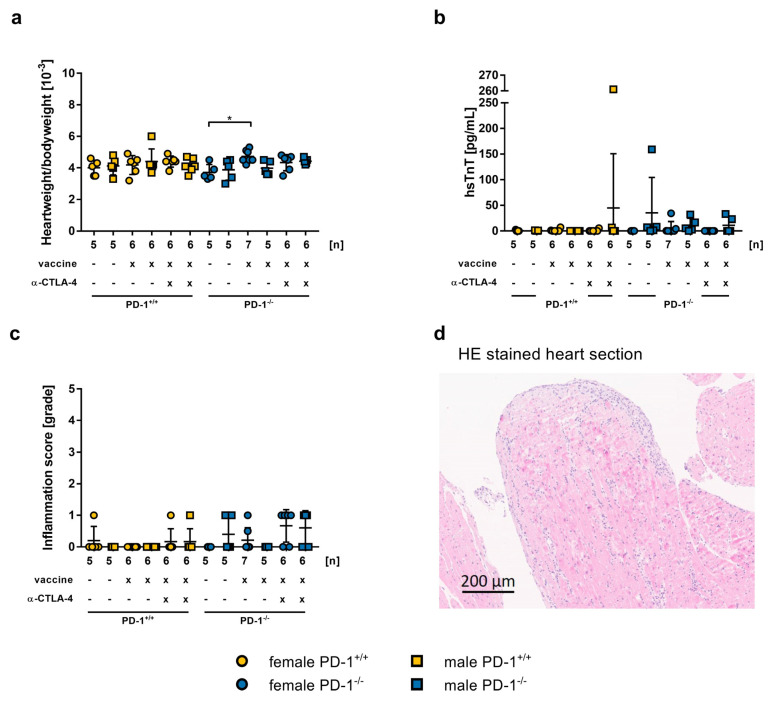
Impact of 2.5 µg mRNA-vaccine on BL/6 PD−1^+/+^ and BL/6 PD-1^−/−^ in combination with immune inhibition. Data is presented as a mean ± SEM (PD−1^+/+^ untreated n = 5, vaccine n = 6, vaccine + aCTLA-4 n = 6; PD-1^−/−^ untreated n = 5, vaccine female n = 7, male n = 5, vaccine + aCTLA-4 n = 6). (**a**) Heart weight to body weight ratio on day 42. Statistical significance was determined by *t*-test, * p = 0.0029. (**b**) Determination hsTnT-levels of mouse serum collected on day 42. (**c**) Histopathological examination of inflammation score in grades in HE stained heart sections on day 42, evaluated by two independent investigators. (**d**) Inflamed heart section of a BL/6 PD-1^−/−^ mouse after vaccination and anti-CTLA-4 co-treatment.

To further investigate the safety of vaccination in conditions representing immune checkpoint blockade treatments, we used PD-1^−/−^ mice and anti-CTLA-4 abs. PD-1^−/−^ mice were vaccinated on day 0 and day 21. One group was additionally treated with CTLA-4 antibodies every three days. Overall, the heart weight to body weight ratio was normal. Only PD-1^−/−^ females treated with mRNA-vaccine showed a significantly higher heart weight to body weight ratio than untreated female mice (4.729 ± 0.152, 3.720 ± 0.220, *p* = 0.0029, respectively; Figure 6a). To determine myocardial damage, hsTnT-levels were measured. We found significantly increased hsTnT-levels in one PD-1^+/+^ male mouse co-treated with mRNA-vaccine and anti-CTLA-4 antibody (261 pg/mL), and in one untreated PD-1^−/−^ mouse (159 pg/mL). All other groups showed no elevated hsTnT-levels (Figure 6b). To determine myocardial inflammation, we performed histopathological analysis of the heart. When untreated, two PD-1^−/−^ mice showed an inflammation score of one. The vaccination by itself induced no inflammation in PD-1^+/+^ mice, but two vaccinated PD-1^−/−^ mice harbored an inflammation score of one. The combination of mRNA-vaccination and anti-CTLA-4 ab resulted in an inflammation score of one in two PD-1^+/+^ mice, and seven PD-1^−/−^ mice (Figure 6c). A representative microscopic picture of a PD-1^−/−^ mouse heart stained with HE is shown in Figure 6d and in Figure S1.

## 3. Discussion

Since the SARS-CoV-2 virus first spread in 2019, much research has been conducted worldwide. To fight back against the virus, which has caused several million deaths, pharma industries developed different types of vaccines, including mRNA technologies against the corona spike protein [6,8,48,49,50]. These are modified mRNAs, delivered by lipid nanoparticles, that encode for the S-protein, which leads to the production of antibodies by presenting the viral protein to endogenous immune cells [51,52,53]. As one of the side effects, the development of myocarditis after the second immunization, especially in young men, was reported [54,55,56,57]. Side effects are constantly reported and collected by the Vaccine Adverse Event Reporting System [58]. It was found that as a side effect, myocarditis is more common in males than in females, and has a high incidence in infants, adolescents, and young adults [59,60,61,62]. If the cases of myocarditis are considered within a range of 12–94 years of age, the mean age of those effects is 26, and symptoms appeared, on average, 3 days after vaccination. Prioritizing the age range of people under 30, the mean age shifts to 19 years, and symptoms occurred after 2 days [63]. The underlying mechanisms have not yet been further elucidated. We tested the BioNTech/Pfizer mRNA vaccine Comirnaty in different gender, ages, and doses to assess the potential age-, gender-, and dose-specific effect by using A/J mice, which is one of the most susceptible strains for induction of autoimmune myocarditis. The histopathological evaluation showed no myocardial inflammation, and deterioration of the cardiac function could not be observed in the echocardiogram, independent of gender, age, and different dosages of the vaccine. In conclusion, our results showed that a double vaccination with mRNA-vaccine is neither causing myocarditis, nor deterioration of cardiac function in these mice (A/J). Furthermore, additional mRNA-vaccination during an ongoing myocarditis induced in these mice, by immunization with cardiac troponin I, did not have a worsening effect on the severity of the induced myocarditis, nor on the cardiac function in this EAM model.

Finally, to study the safety and risks of SARS-CoV-2 mRNA-vaccines in ICI co-treatment conditions, we assessed the effects of vaccination in PD-1^−/−^ mice, and in PD-1^−/−^ mice additionally co-treated with anti-CTLA-4 abs. In recent years, treatment with ICIs has steadily improved, and the use of antibodies against PD-1, PD-L1, and CTLA-4 alone, as well as in combinations, has achieved promising results in the treatment of cancer patients. However, cardiovascular immune-related adverse events (irAEs) occur in 1.4–5% of cases. Among these events, myocarditis, with an incidence of approximately 0.09% to 1.80%, is one of the most common irAEs in an early stage of treatment, but is a less common side effect overall. Despite this, myocarditis has a high mortality rate of up to 50% [64,65,66]. Still, the safety and side effects of SARS-CoV-2 vaccination for patients undergoing ICI treatment are not known. It was reported that cancer patients have a higher risk for developing severe COVID when they were treated with ICI (reviewed in [67]). Further investigation into SARS-CoV-2 vaccination in combination with immunotherapy is necessary. Waissengrin et al. reported, in a study with cancer patients who received ICI treatment and a full immunization with BioNTech mRNA-vaccine, a similar side effect profile compared to healthy controls, and no additional irAEs. This study was based on a small cohort of 134 patients, due to lack of suitable participants [68]. However, our results showed that some mice had significantly elevated troponin T values in the mRNA-vaccine and CTLA-4 abs co-treated group, as well as in the PD-1^−/−^ untreated group. Furthermore, we found several mice which developed a myocardial inflammation, though the severity of the inflammation was rather low. Therefore, it seems to be important to monitor these groups of patients after SARS-CoV-2 vaccination more carefully, regarding cardiac markers and function. Further basic and clinical studies are needed to obtain more information on the safety of vaccination in this subgroup of patients with ongoing myocarditis, or other risk factors, such as ICI treatment.

## 4. Materials and Methods

### 4.1. Animals

Male (m) and female (f) A/JOlaHsd (A/J) and C57BL/6 (BL/6) mice, as well as the C57BL/6 PD-1 knockout (PD-1^−/−^) mice (B6.Cg-Pdcd1tm1.1Shr/J), were used. In the experiments on the dose-, age-, and gender-specific effect in A/J and BL/6 mice, as well as in the experiments on the impact of ICI treatment in BL/6 and BL/6 PD-1^−/−^ mice, animals from our own breeding were used. This resulted in a larger variance of the n-number. A/J mice for the experimental approach for parallel induction of myocarditis were obtained from Envigo (Huntington, Cambridgeshire, UK). All mice were kept in the animal facility of the University of Heidelberg. Based on the Directive of the European Parliament and the Council for the protection of animals used for scientific purposes, this study was conducted in accordance with the German animal welfare act (Directive 2010/63/EU). The local Animal care and use Committee in Karlsruhe approved all procedures, including the use and care of animals (approval reference number G-4/21).

### 4.2. Treatment and Induction of Experimental Autoimmune Myocarditis

To study the vaccine in mouse strains with different susceptibilities for EAM [31], and to study the effect of age and gender, male and female A/J (5–7 or 23-weeks) or BL/6 mice (4–5 weeks) were treated with an intramuscular injection of 0.2 μg or 2.5 μg BioNTech SARS-CoV-2 mRNA-vaccine (Pfizer Pharma GmbH, Berlin, Germany) on day 0 and day 21. mRNA-vaccine was left over from internal staff vaccination that had not further been used, and otherwise would have been disposed of. To investigate the effect of vaccination with 2.5 µg mRNA-vaccine on the induction of EAM, male and female A/J mice at the age of 4–5 weeks were additionally subcutaneously immunized three times, at 7-day intervals (day 0, 7, 14), with 150 µg murine cardiac troponin I (mcTnI) peptide (Peptide Specialty Laboratories GmbH, Heidelberg, Germany) in supplemented complete Freud’s adjuvant with 5 mg/mL *Mycobacterium tuberculosis* H37Ra (Sigma, St. Louis, MO, USA) [33]. To test the effect of vaccination on novel immunotherapy (inhibition of PD-1/CTLA-4), 4–5 weeks old female and male BL/6 wildtype and PD-1^−/−^ mice were vaccinated with 2.5 μg mRNA-vaccine on day 0 and day 21. A proportion of this group was additionally treated with 200 µg anti-CTLA-4 abs (Bio x cell Inc., Lebanon, NH, USA) every three days for 12 times, starting on day 0. As control groups, untreated mice were used. In all experimental setups, blood was taken retrobulbar under inhalation anaesthesia of 5 vol.% isoflurane, with an oxygen flow of 0.8 L/min, in a whole-body chamber on day 0 and day 21, and cardiac function was examined by echocardiography on day 0, 21, and 42. All animals were sacrificed after 42 days by cervical dislocation under intraperitoneal anaesthesia with 120 mg/kg ketamine and 16 mg/kg xylazine (Bermer Pharma GmbH, Warburg, Germany, Ecuphar GmbH, Greifswald, Germany), to collect the blood and heart for further analysis.

### 4.3. Echocardiographic Examination of Cardiac Heart Function

Echocardiographic measurements of the mice were performed on day 0, 21, and 42 using the Visual Sonics Vevo 2100 system, 30 MHz linear MicroScan transducer (MSH400). The long axis was recorded with projection cine loops in order to visualise the cardiac walls and the aortic annulus. Suitable software provided by the Vevo2100 platform was used to determine the left ventricular ejection fraction.

### 4.4. Determination of High-Sensitive Troponin T Levels in Serum

For evaluation of cardiac troponin T as a marker for cardiac damage, high-sensitive troponin T (hsTnT) levels were determined in serum of the mice. For this purpose, serum was diluted 1:10 with NaCl (0.9%) (Braun AG) and measured via an ELECSYS 2010 automated analyzer (Roche Diagnostics, Mannheim, Germany). This method has a blank limit of 3 pg/mL and a detection limit of 5 pg/mL [69]. Due to our dilution, values > 50 pg/mL are pathological.

### 4.5. Histopathological Examination of Inflammation and Fibrosis

Hearts were cut in half along the longitudinal axis, vertical to the septum. One half was fixed in 10% formalin for at least 24 h. The hearts were embedded in paraffin and cut into 2–3 µm thick sections, which were subsequently stained by haematoxylin and eosin (HE) using standard protocols and reagents. Inflammation scores were determined via light microscopy by two independent observers who were blinded to the treatment and immunization status of the animals and groups. Inflammation scores are indicated as percentage in relation to the whole heart sections, and calculated as means from the values of both investigators. The observed severity of inflammation was then classified into groups from 0 to 5. The grading of the inflammation score is shown in Table 1.

### 4.6. Detection of Antibodies against Spike Protein Peptide Sequences via Enzyme-Linked Immunosorbent Assay (ELISA)

Serum blood samples were collected from each mouse, and antibody titers were determined by using the ELISA technique. These were performed as previously described [70,71]. Therefore, 96-well microtiter plates (Thermo Fisher Scientific Inc., Waltham, MA, USA) were coated with 100 µL/well of pooled spike peptide sequences in bicarbonate buffer (pH 9.6), and incubated overnight. Mouse secondary IgG antibodies were diluted to 1:5000 and used for detection. Serum samples were diluted for pooled spike peptides to 1:10, 1:20, 1:40, 1:50, 1:80, 1:200, 1:320; 1:800, and 1:3200. Normal mouse serum was used as control. The color reaction was developed with TMB (KPL) (Surmodics IVD, Inc., Eden Prairie, MN, USA), and stopped by adding 100 μL of 0.3M H_2_SO_4_ (Honeywell Holding GmbH) to each well. Optical densities were determined at 450 nm. Antibody endpoint titers for each individual mouse were calculated as the greatest positive dilution of antibody.

### 4.7. Comparative Values for All Measured Parameters Analyzing Myocarditis and Their Corresponding References Are Listed in Table S1

For easier comparison, we collected some references for all measured parameters regarding myocarditis and their corresponding references in a Supplementary Table (Table S1).

### 4.8. Statistical Analysis and Graphical Representation

All graphs were plotted, and statistical analysis was performed using GraphPad Prism version 7.00 for Windows (GraphPad Software, La Jolla, CA, USA), with data plotted as individual points shown as mean ± standard error of the mean. The values were tested for normality using the D’Agostino–Pearson normality test. Groups were analyzed using *t*-test for parametric data. For the comparison of multiple measurements of different groups, ANOVA was used, followed by a multiple comparison test. All tests used were 2-tailed. The threshold for significance was set at 0.05.

## Figures and Tables

**Table 1:** Overview of the determined inflammation score in grades and percent.

Inflammation Score [Grade]	Inflammation [%]
0	0
1	1–20
2	21–40
3	41–60
4	61–80
5	>80

## Data Availability

The data presented in this study are available on request from the corresponding author.

## Supplementary Materials

The following supporting information can be downloaded at: https://www.mdpi.com/article/10.3390/ijms24055011/s1. References [72,73,74,75] are cited in the Supplementary Materials.
