# Supplementary material for: Effect of SARS-CoV-2 mRNA-Vaccine on the Induction of Myocarditis in Different Murine Animal Models
Source: Int J Mol Sci. 2023 Mar 6;24(5):5011. doi: 10.3390/ijms24055011 (PMC10002951; doi:10.3390/ijms24055011)
Supplement: Supplementary file 1 [file ijms-24-05011-s001.zip › ijms-2190360-supplementary.pdf]

Supplementary Material

Effect of SARS-CoV-2 mRNA-vaccine on the induction of myocarditis in different murine animal models

Vanessa A. Zirkenbach, Rebecca M. Ignatz, Renate Öttl, Zeynep Cehreli, Vera Stroikova, Mansur Kaya, Lorenz H. Lehmann, Michael R. Preusch, Norbert Frey, Ziya Kaya

Supplementary Tables

Supplementary Figures

Supplementary Tables:

Table S1: Overview of normal and pathological values of different parameters used to evaluate myocarditis. Some of these parameters are depending on the used mouse strain and age, as well as on the method utilized for determination.

Parameter	Normal condition	Anomaly	References
hsTnT	<50 pg/ml (lower detection limit)	≥50 pg/ml	[35,69,72]
Ejection fraction	60-90% (dependent on mouse strain)	≤60% (dependent on mouse strain)	[35,36,73,74]
Inflammation	Score = 0	Score ≥1	[32, 33, 40]
Antibody titer	<1:10 (lowest dilution used in this study)	≥1:10	[32,33, 45-7,73,75,]

Supplementary Figures

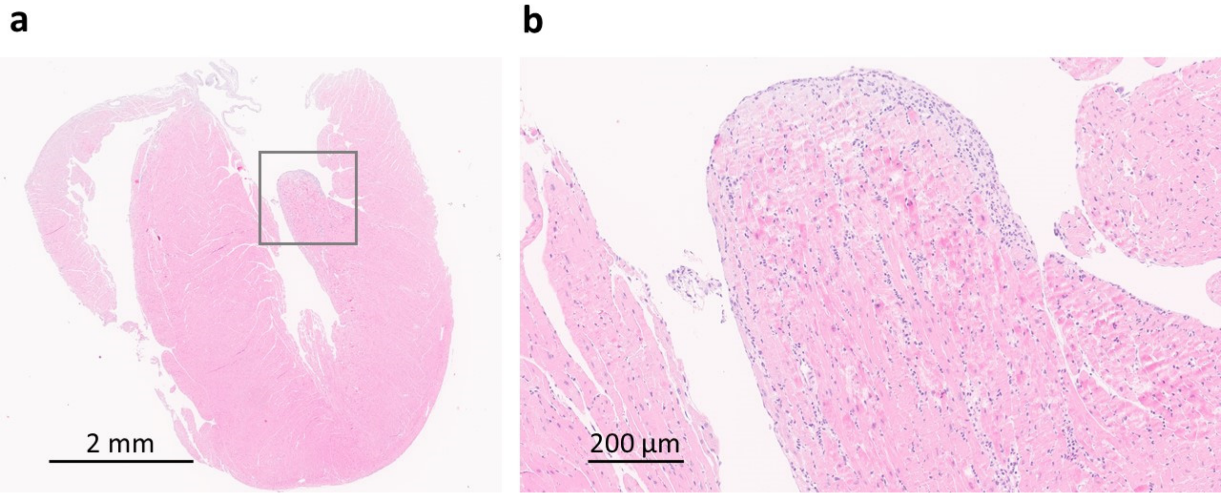

Figure S1: Inflamed heart section of a BL/6 PD-1$^{-/-}$ mouse after vaccination and anti-CTLA-4 co-treatment stained with Hematoxylin-eosin in **a)** 1,5x magnification and **b)** 8x magnification. Square in a) showing the region for b).
